# Gut Microbial Characteristics of Adult Patients With Epilepsy

**DOI:** 10.3389/fnins.2022.803538

**Published:** 2022-02-16

**Authors:** Lian Dong, Qian Zheng, Yongran Cheng, Mengyun Zhou, Mingwei Wang, Jianwei Xu, Zucai Xu, Guofeng Wu, Yunli Yu, Lan Ye, Zhanhui Feng

**Affiliations:** ^1^Department of Neurology, Affiliated Hospital of Guizhou Medical University, Guiyang, China; ^2^School of Public Health, Hangzhou Medical College, Hangzhou, China; ^3^Department of Molecular and Cellular Physiology, Shinshu University School of Medicine, Nagano, Japan; ^4^Department of Cardiology, Affiliated Hospital of Hangzhou Normal University, Hangzhou, China; ^5^National Guizhou Joint Engineering Laboratory for Cell Engineering and Biomedicine Technique, Guizhou Province Key Laboratory of Regenerative Medicine, Center for Tissue Engineering and Stem Cell Research, Guizhou Medical University, Guiyang, China; ^6^Department of Neurology, Affiliated Hospital of Zunyi Medical University, Zunyi, China; ^7^The Medical Function Laboratory of Experimental Teaching Center of Basic Medicine, Guizhou Medical University, Guiyang, China

**Keywords:** epilepsy, microbiota-gut-brain axis, gut microbiota, 16S rRNA, biomarkers, PICRUSt

## Abstract

**Objective:**

To characterize the intestinal flora of patients with epilepsy and its correlation with epilepsy.

**Methods:**

Patients with ages > 18 years were consecutively enrolled from the outpatient department, Affiliated Hospital of Guizhou Medical University from January 2018 to December 2019. A total of 71 subjects were recruited, including epilepsy patients (*n* = 41) as an observation group and patient family members (*n* = 30) as a control group. Fresh stool specimens of all the subjects were collected. The 16S ribosomal RNA sequencing was analyzed to determine changes in intestinal flora composition and its correlation with epilepsy. Subgroup analysis was then conducted. All patients with epilepsy were divided into an urban group (*n* = 21) and a rural group (*n* = 20) according to the region, and bioinformatics analyses were repeated between subgroups.

**Results:**

LEfSe analysis showed that *Fusobacterium*, *Megasphaera*, *Alloprevotella*, and *Sutterella* had relatively increased abundance in the epilepsy group at the genus level. Correlation analysis suggested that *Fusobacterium* sp. (*r* = 0.584, *P* < 0.01), *Fusobacterium mortiferum* (*r* = 0.560, *P* < 0.01), *Ruminococcus gnavus* (*r* = 0.541, *P* < 0.01), and *Bacteroides fragilis* (*r* = 0.506, *P* < 0.01) were significantly positively correlated with the occurrence of epilepsy (*r* ≥ 0.5, *P* < 0.05). PICRUSt function prediction analysis showed that there were significant differences in 16 pathways between the groups at level 3. Comparing the rural group with the urban group, *Proteobacteria* increased at the phylum level and *Escherichia coli*, *Fusobacterium varium*, *Prevotella stercorea*, and *Prevotellaceae bacterium DJF VR15* increased at the species level in the rural group.

**Conclusion:**

There were significant differences in the composition and functional pathways of gut flora between epilepsy patients and patient family members. The *Fusobacterium* may become a potential biomarker for the diagnosis of epilepsy.

## Introduction

Epilepsy is one of the most common and serious nervous system diseases, affecting more than 70 million people worldwide. There are more than 9 million epilepsy patients in China, with approximately 650,000 new epilepsy patients diagnosed each year (Ngugi et al., 2011). Approximately a third of the patients have drug-resistant epilepsy (Tang et al., 2017). The pathogenesis of epilepsy is multifactorial, and the specific mechanisms remain largely unknown.

Gut microbes could regulate brain function and behavior through the brain–gut axis (Johnson and Foster, 2018). The brain–gut axis was involved in bidirectional regulation between gastrointestinal functions and the central nervous system. Gut microbiota and their metabolites, such as short chain fatty acids, lipopolysaccharides (LPSs), and amino acids, affected brain function through the brain-gut axis. However, the brain can also affect the gut microbes through the axis by regulating the secretion of intestinal peptides, gastric acid and mucus, intestinal permeability and mucosal immunity through the autonomic nervous system which affect the habitat of intestinal microorganisms, thus affecting the composition of intestinal microbes. The neuroendocrine system can directly affect the intraluminal release of 5-HT, catecholamines and cytokines from enterochromaffin cell, immune cells and neurons on the other hand (Martin et al., 2018).

There is a lot of research showed that inflammation plays an important role in the pathophysiology of epilepsy (Aronica et al., 2017). Gut microbes and its secretion can induce the increase of inflammatory cytokines such as IL-6 and IL-1 in microglia (Xu et al., 2021), it can also destroy the paracellular barrier via epithelial e-cadherin, which resulting in the increase of blood-brain barrier permeability, and then reducing the response threshold of neurons and improving the excitability of neurons (Lukiw, 2020). Certain intestinal flora could also secrete neurotransmitters such as dopamine, norepinephrine, 5-hydroxytryptamine, acetylcholine, and GABA, which directly or indirectly affected the excitability of the central nervous system (Jenkins et al., 2016; Mittal et al., 2017). Therefore, gut microbes may affect the occurrence and progress of epilepsy via the regulation of inflammatory cytokine and neurotransmitter levels.

A clinical case-control study showed that the levels of *Verrucomicrobia* and *Actinobacteria* in the intestinal tract of epileptic patients were higher than those of normal people at the phylum level (Gong et al., 2020). At the genus level, the levels of *Romboutsia*, *Megamonas*, and *Bifidobacterium* were significantly higher than those of normal humans, while the levels of *Sutterella* and *Klebsiella* were significantly lower (Gong et al., 2020). It meant that levels of *Bifidobacterium longum*, *Enterococcus faecium*, and *Eggerthella lenta* could be used as biomarkers for refractory epilepsy. One study reported that at the phylum level, the level of *Proteobacteria* in the gut of epilepsy patients was significantly increased, compared with the control group, and the levels of *Neisseria*, *Haemophilu*s, *Delftia*, and *Lautropia* were significantly increased at the genus level (Şafak et al., 2020). Citraro et al. (2021) first characterized the intestinal flora of absence seizure rats (WAG/Rij rats). They found that WAG/Rij rats had a significant difference in intestinal flora, when compared with the normal rats when they were 4-months-old, which mainly involved increased abundances of *Clostridiales*, *Clostridiaceae*, and *Lachnospiraceae*, and decreased abundances of *Lactobacillus* and *Phascolarctobacterium* (Citraro et al., 2021). In addition, they found that there already was intestinal villi disruption and inflammatory infiltrates when WAG/Rij rats were 1 month of age, indicating that gut microbiota and epilepsy were interconnected.

Medel-Matus et al. (2018) showed that the chronic stress could result to the change of intestinal flora of the rats, and transplantation of intestinal flora of sham-stressed rats into the intestine of chronic stressed rats can partially resist the epileptic effect of chronic stress, and conversely it can increase the susceptibility to epilepsy of sham-stressed rats. Another study showed that fecal microbiota transplantation (FMT) could change the composition of intestinal flora. It reported that a child with Crohn’s disease and epilepsy had no seizures in the 20 months of follow-up the FMT treatment (He et al., 2017).

In this study, we analyzed the intestinal microbial composition of epilepsy patients and their families based on 16S ribosomal RNA sequencing, and used the random forest prediction model to identify biomarkers for epilepsy diagnosis, to further predict the functional pathways in the gut of epilepsy patients, through which intestinal microbial and epilepsy were linked, so as to provide a new strategy for epilepsy diagnosis and treatment.

## Materials and Methods

### Study Participants and Sample Collection

Our study was approved by the Ethics Committee of the Affiliated Hospital of Guizhou Medical University. The approval number was 146.

Patients with epilepsy and their accompanying family members who were examined in the electroencephalogram room or hospitalized in our hospital from November 2018 to June 2020 were divided into the epilepsy and control groups.

The enrollment criteria of the epilepsy group were the following: (1) epilepsy patients who met the 2017 ILAE criteria for epilepsy (Fisher et al., 2017) (all types of epilepsy were considered, including focal, general, structural, infectious, immune and hereditary); (2) the patients and their family members lived in the same region; and (3) an age > 18 years. The exclusion criteria were the following: (1) patients who were treated with antibiotics or probiotics (or prebiotics) within 3 months; (2) patients with clear histories of chronic diseases, such as hypertension, diabetes, inflammatory bowel disease, irritable bowel syndrome, and other gastrointestinal diseases; and (3) patients who were complicated with other neurological or psychiatric diseases, such as anxiety, depression, Parkinson’s disease, and Alzheimer’s disease. The enrollment criteria of the control group involved: (1) families of the epilepsy patients as the control group, who lived in the same area with the patients; (2) those who never experienced seizures in their life; and (3) an age older than 18 years. The exclusion criteria were the same as those of the epilepsy group. We have made a survey about type of meal for all the members of families. The control group, coming from the families, has more consistent eating habits with patients.

A total of 71 subjects were continuously enrolled in our study, containing the epilepsy group (*n* = 41) and the control group (*n* = 30). All subjects signed informed consent forms. The participants’ clinical data were recorded, including gender, age, body mass index, ethnicity, residential area (rural or urban), frequency of seizures, course of disease, and use of anti-epileptic drugs. Fresh feces (approximately 200 mg) were sampled using a sampling box (including storage box and cotton swabs, which were all sterile) produced by Omega Bio-Tek (Norcross, GA, United States), and stored at –20°C.

### Study Design

We analyzed and compared the composition of the intestinal flora between the epilepsy and control groups using 16S rRNA analyses, and further investigated the composition and functional pathway differences of the intestinal flora between the two groups using diversity analyses, differential species analyses, random forest analyses, and function predictions. As higher seizure frequencies of epilepsy in rural areas than patients from urban groups (UG) based on our baseline data. In the second part, we conducted a subgroup analysis, and determined the composition of the intestinal flora between the epilepsy patients from rural and urban areas using diversity analysis.

### Gene Sequencing

After sample collection, the intestinal flora DNA was extracted, using a PowerSoil^®^ DNA Isolation Kit (Qiagen, Hilden, Germany), PCR amplification, and sequencing by Shanghai Biotree Biotech (Shanghai, China). V3–V4 regions of the 16S rRNA were amplified by the 515F primer (5′-ACTCCTACGGGAGGCAGCA-3′) and 806R (5′-GGACTACHVGGGTWTCTAAT-3′). PCR products were detected by electrophoresis with 2% agarose gels. A TruSeq^®^ DNA PCR-Free Sample Preparation Kit (Illumina, San Diego, CA, United States) was used for construction of a genomic library. The constructed library was quantified by qubit and Q-PCR, and then sequenced by NovaSeq PE250 (Illumina).

### Bioinformatics Processing

For the raw sequences obtained by the Illumina NovaSeq, we performed sequence assembly, filtration, and constructions of chimeras according to the Qiime (version V1.9.1)^1^ quality control process, and then we obtained the effective data for subsequent analysis. All effective data of the samples were clustered into operational taxonomic units (OTUs) at 97% identity using Uparse Software (version 7.0.1001).^2^ The Mothur method and the SSUrRNA database of SILVA132 were used to perform a species annotation on the OTU sequences. Finally, the sequencing data were rarefied.

The species accumulation boxplot and rarefaction curve showed that the sample size was sufficient. The within-diversity of the sample (i.e., α-diversity) was calculated. The inter-sample diversity (i.e., β-diversity) was determined based on the weighted and unweighted Unifrac, and was visualized by principal coordinate analysis (PCoA), which reduced the dimensions of the distance matrix into a two-dimensional graph. The Wilcoxon rank sum test was used for statistical analysis between both groups.

Linear discriminant effect size analysis (LEfSe) showed the species with significant differences in abundance among different groups, and identified the intestinal flora with the most biological characteristics, and then calculated the logarithmic linear discriminant analysis (LDA) score (LDA ≥ 3.0 are generally considered to be discriminative).

We constructed a random forest model (including the training and test models) based on the relative abundance of intestinal flora of the top 10 at the genus level. MeanDecreaseGin was used to identify important species, followed by cross-validation, constructing a receiver operating characteristic curve, and calculating the area under the curve (AUC) to evaluate the performance of the random forest model.

The phylogenetic investigation of communities assessed by reconstruction of unobserved states (PICRUSt) was used for predictions of functions. It inferred gene function profiles based on the gene information on the OTU in the Greengene database, and inferred the gene function profiles of other untested species at the same time. We then constructed a gene function prediction profile of the entire bacterial spectrum, finally “mapped” the sequenced flora data to the database, and performed Kyoto Encyclopedia of Genes and Genomes pathway annotations.

### Statistical Analysis

SPSS statistical software for Windows, version 24.0 (SPSS, Chicago, IL, United States) was used for analysis of the baseline data. Two group means were compared using Student’s *t*-test and categorical variables were analyzed using the chi-square test. Measurement data are expressed as the mean ± standard deviation (mean ± SD). Spearman’s correlation was used for analysis of the relationship between intestinal flora and epilepsy. A value of *P* < 0.05 was considered significant. The correlation coefficient |r| between 0.8 and 1.0 meant a very strong correlation, 0.6–0.8 meant a strong correlation, 0.4–0.6 meant a moderate correlation, 0.2–0.4 meant a weak correlation, and 0.0–0.2 meant a very weak or no correlation.

## Results

### Demographic Characteristics of the Study Population Between the Epilepsy and Control Groups

A total of 41 cases in the epileptic group (EG) and 30 cases in the control group (CG) were enrolled. To decrease the influence of diet on intestinal flora, the control groups were from the families of patients. There was no statistical difference between the EG and CG in average age (31.6 and 34.6 years, respectively), sex (males: 56.1%, and females: 33.3%, respectively), body mass index (BMI) (22.5 and 23.3, respectively), race (Han: 59.5% and other: 60.0%, respectively), place of residence (urban: 51.2 and 46.7%, respectively), and living habits (i.e., smoking, drinking) (*P* > 0.05). Among patients with epilepsy, 84.4% involved focal onset. In terms of etiology, 29.3% were structural, 12.2% were infectious, 2.4% were immune, and 56.1% were of unknown origin (Table 1).

**TABLE 1:** Demographic characteristics of the study population between the epileptic and control groups.

	EG (*n* = 41)	CG (*n* = 30)	*P*-value
Age (year)	31.6 ± 12.2	34.6 ± 7.6	0.206
**Sex**			
Male	23 (56.1%)	10 (33.3%)	0.057
Female	18 (43.9%)	20 (66.7%)	
BMI	22.5 ± 4.1	23.3 ± 2.7	0.328
**Race**			
Han	24 (59.5%)	18 (60.0%)	0.901
Others	17 (41.5%)	12 (40.0%)	
**Place of residence**			
Urban	21 (51.2%)	14 (46.7%)	0.705
Rural	20 (48.8%)	16 (53.3%)	
**Smoking**			
Yes	13 (31.7%)	7 (23.3%)	0.438
Not	28 (68.3%)	23 (76.7%)	
**Drinking**			
Yes	6 (14.6%)	6 (20.0%)	0.551
Not	35 (85.4%)	24 (80.0%)	
Age of onset (year)	22.1 ± 14.6	−	−
course of disease (year)	7.5 ± 7.8	−	−
Seizure type			
Focal onset	35 (84.4%)	−	−
Generalized onset	6 (14.6%)		
**Etiology**			
Structural	12 (29.3%)	−	−
Infectious	5 (12.2%)		
Immune	1 (2.4%)		
Unknown	23 (56.1%)		
**Frequency (seizures per year)**			
0–4	18 (46.2%)	−	−
5–12	7 (17.9%)		
13–52	8 (20.5%)		
>52	6 (15.4%)		
**Number of ASMs**			
1	21 (61.8%)	−	−
2	10 (29.4%)		
3	3 (8.8%)		

*Continuous variables were expressed as mean ± standard deviation, and categorical variables were expressed as percentages.*

### Characteristics of Intestinal Flora in Patients With Epilepsy

**FIGURE 1:**
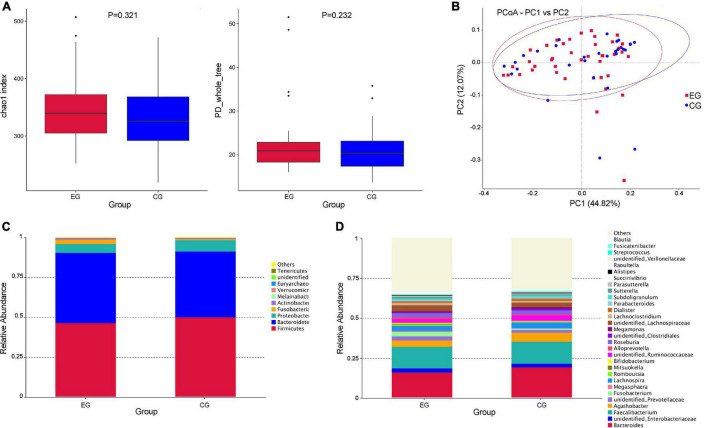
Diversity analysis of intestinal flora and the relative abundance of microbes in the epileptic group (EG) and control group (CG). **(A)** The α diversity (Chao1 and PD_whole_tree indices) showed that there was no significant difference in diversity of gut microbe between the EG and CG (*P* > 0.05, *P*-values were 0.321 and 0.232, respectively, calculated using the Wilcoxon rank sum test). **(B)** The PCoA analysis of β diversity based on the weighted UniFrac distance showed that the composition of intestinal microbes in the EG was different from that in the CG (EG is expressed by red squares and CG is expressed by blue dots). **(C)** The relative abundances of the top 10 microbes at the phylum level in the EG and CG. **(D)** The relative abundances of the top 30 microbes at the genus level in the EG and CG.

The α-diversity of the EG was slightly higher (the chao1 index of the EG was 345.780, the Shannon index was 0.925, the chao1 index of the CG was 332.142, and the PD_whole_tree index was 0.920) than the CG, but there was no statistical difference (*P* > 0.05; the *P*-values were 0.321 and 0.232, respectively, calculated using the Wilcoxon rank sum test) (Figure 1A). PCoA analysis of β-diversity based on the weighted UniFrac distance showed that the composition of intestinal microbes in the EG was different from that in the CG (Figure 1B).

At the phylum level, *Firmicutes* was the largest phylum of the EG and CG, but the abundance in the EG was relatively lower than that in the CG (46.6% EG and 50.3% CG), and the *Bacteroides* was the second largest phylum (44.0% EG and 41.1% CG). The abundance of *Fusobacteria* showed a significant increase in EG (2.8% EG and 0.8% CG). Some rare microbes had an increased trend in EG, including *Verrucomicrobia* (0.07% EG and 0.02% CG) and *Actinobacteria* (0.9% EG and 0.5% CG) (Figure 1C). At the genus level, *Fusobacterium* (2.7% EG and 0.8% CG), *Megasphaera* (0.54% EG and 0.09% CG), *Alloprevotella* (1.3% EG and 0.8% CG), *Sutterella* (1.0% EG and 0.5% CG), and *Streptococcus* (2.0% EG and 0.04% CG) had higher abundance in the EG (Figure 1D).

**FIGURE 2:**
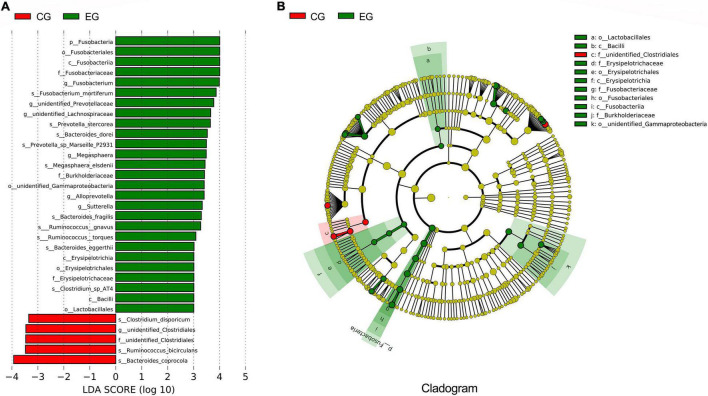
The difference analyses of intestinal microbes between the epileptic group (EG) and control group (CG). **(A)** LEfSe analysis; the length of the bar is the logarithm of the linear discriminant analysis (LDA). Green bars indicate microbes enriched in the EG; red bars indicate microbes enriched in the CG (only species with an LDA ≥ 3.0 are shown). **(B)** The phylogenetic tree expressed as a cladogram. The green nodes indicate microbes enriched in the EG, the red nodes indicate microbes enriched in the CG.

The LEfSe analysis further confirmed the differences of intestinal microbes between EG and CG. In the EG, the abundance of *Fusobacteria* was significantly increased at the phylum level, the abundance of *Fusobacterium, Megasphaera*, *Alloprevotella*, and *Sutterella* were relatively higher at the genus level, and the abundance of *Fusobacterium mortiferum*, *Prevotella stercorea, Bacteroides dorei*, *Prevotella* sp. *Marseille P2931*, *Megasphaera elsdenii*, *Bacteroides fragilis*, *Ruminococcus gnavus*, *Ruminococcus torques*, *Bacteroides eggerthii*, and *Clostridium* sp. *AT4* were increased at the species level, while the abundance of *Clostridium disporicum*, *Ruminococcus bicirculans*, and *Bacteroides coprocola* were decreased compared with the CG (Figures 2A,B).

### Correlation Analysis Between Intestinal Microbes and Epilepsy

Spearman’s correlation analysis showed that the abundance of *Fusobacterium mortiferum*, *Bacteroides fragilis*, *Ruminococcus gnavus*, and *Fusobacterium* were positively correlated with epilepsy (Table 2).

**TABLE 2:** The correlation analysis between intestinal microbe and epilepsy.

Microbes	*r*-value	*P-*value
*Fusobacterium*	0.584	0.000
*Fusobacterium mortiferum*	0.560	0.000
*Ruminococcus gnavus*	0.541	0.000
*Bacteroides fragilis*	0.506	0.000
*Alloprevotella*	0.466	0.000
*Prevotella* sp. *Marseille* P2931	0.426	0.000
*Megasphaera*	0.387	0.001
*Clostridium* sp. AT4	0.370	0.002
*Megasphaera elsdenii*	0.367	0.002
*Bacteroides dorei*	0.351	0.003
*Clostridium disporicum*	–0.305	0.010
*Bacteroides coprocola*	–0.325	0.006

### Random Forest Analysis

**FIGURE 3:**
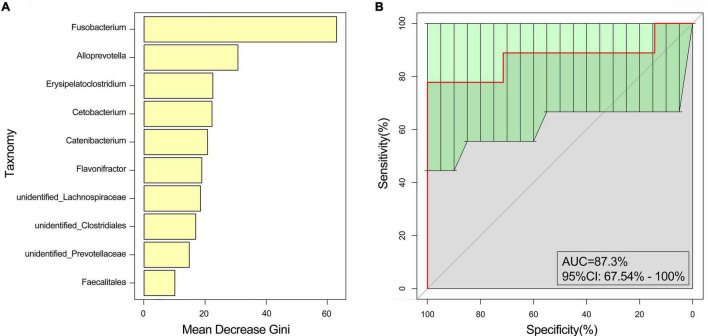
Random forest analysis. The marked species were screened by MeanDecreaseGin. **(A)** MeanDecreaseGin analysis at the genus level; abscissa: average decline of the Gini index; ordinate: intestinal microbes of the top 10. **(B)** Receiver operating characteristic curve analysis was used to evaluate the performance of the random forest.

In our study, we established two models, the training model (*n* = 33 patients and *n* = 24 healthy controls) and the test model (*n* = 8 patients and *n* = 6 healthy controls). At the genus level, *Fusobacterium* played an important role in distinguishing between the EG and CG [training model: AUC: 94.43%, 95% confidence interval (CI): 88.7–100%; test model: AUC: 87.3%, 95%CI: 67.54–100%] (Figures 3A,B).

### Functional Predictions of Intestinal Microbes in the Epileptic Group and Control Group

**FIGURE 4:**
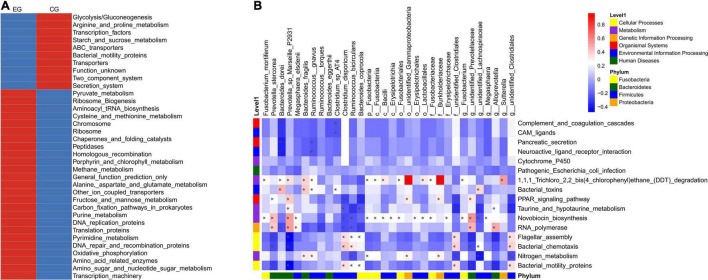
PICRUSt prediction of intestinal microbe function in the epileptic group (EG) and control group (CG). **(A)** The relative abundance cluster analysis of the functional pathway shows the top 35 functional pathways in the EG and CG. **(B)** Correlation heat map of differential bacteria and differential functional pathways (*P < 0.05).

PICRUSt was used for function predictions. At the third level, there was mainly enrichment of amino sugars and nucleotide sugar metabolism, oxidative phosphorylation, and fructose and mannose metabolism, and alanine, aspartate, and glutamate metabolism were enriched in the EG, while the secretion system, ABC transporter, starch and sucrose metabolism, glycolysis/gluconeogenesis, arginine, and proline metabolism were enriched in the CG (Figure 4A).

The pathways of Bacterial motility proteins (0.99 ± 0.22% vs. 1.16 ± 0.28% in the EG and CG, respectively, *P* = 0.006), bacterial chemotaxis (0.73 ± 0.30% vs. 0.71 ± 0.20%, *P* = 0.037), flagellar assembly (0.53 ± 0.12% vs. 0.60 ± 0.14%, *P* = 0.004), nitrogen metabolism (0.42 ± 0.10% vs. 0.51 ± 0.12%, *P* = 0.044), novobiocin biosynthesis (0.15 ± 0.01% vs. 0.14 ± 0.02%, *P* = 0.025), taurine and hypotaurine metabolism (0.14 ± 0.00% vs. 0.13 ± 0.01%, *P* = 0.039), 1,1,1-trichloro-2,2-bis (4-chlorophenyl) ethane (DDT) degradation (0.11 ± 0.01% vs. 0.10 ± 0.01%, *P* = 0.008), cytochrome P450 (0.11 ± 0.01% vs. 0.10 ± 0.01%, *P* = 0.038), RNA polymerase (0.10 ± 0.01% vs. 0.09 ± 0.01%, *P* = 0.025), the PPAR signaling pathway (0.00 ± 0.00% vs. 0.00 ± 0.00%, *P* = 0.030), pancreatic secretion (0.00 ± 0.00% vs. 0.00 ± 0.00%, *P* = 0.024), complement and coagulation cascades (0.00 ± 0.00% vs. 0.00 ± 0.00%, *P* = 0.033), bacterial toxins (0.00 ± 0.00% vs. 0.00 ± 0.00%, *P* = 0.019), neuroactive ligand-receptor interaction (0.00 ± 0.00% vs. 0.00 ± 0.00%, *P* = 0.026), CAM ligands (0.00 ± 0.00% vs. 0.00 ± 0.00%, *P* = 0.033), and pathogenic *Escherichia coli* infection (0.00 ± 0.00% vs. 0.00 ± 0.00%, *P* = 0.047), were significantly different between the EG and CG.

Analysis of the relationship between differential microbes and functional pathways indicated that these functional pathways may be associated with *Prevotella* sp. *Marseille P2931, Burkholderiaceae*, *Bacteroides coprocola*, and other bacteria (Figure 4B).

### Demographic Characteristics of the Study Population Between the Urban Group and Rural Group

The epileptic group was divided into the UG and RG according to the place of residence. Statistical analysis showed that there were statistical differences between the two groups in sex, seizure frequency, and the number of medications (*P* < 0.05), but there was no statistical difference in average age, BMI, living habits (smoking, ± drinking), course of the disease, and compliance of taking medication (*P* > 0.05) (Table 3).

**TABLE 3:** Demographic characteristics of the study population between the UG and RG.

	EG (*n* = 41)		*P*-value
	UG (*n* = 21)	RG (*n* = 20)	
Age (year)	34.0 ± 13.7	29.1 ± 10.2	0.207
**Sex**			
Male	8 (38.1%)	15 (75.0%)	0.017
Female	13 (61.9%)	5 (25.0%)	
BMI	22.5 ± 3.9	22.5 ± 4.4	0.972
**Race**			
Han	13 (61.9%)	11 (55.0%)	0.654
Others	8 (38.1%)	9 (45.0%)	
**Place of residence**			
Urban	5 (23.8%)	8 (40.0%)	0.265
Rural	16 (76.2%)	12 (60.0%)	
**Smoking**			
Yes	4 (19.0%)	2 (10.0%)	0.706
Not	17 (81.0%)	18 (90.0%)	
Age of onset (year)	25.7 ± 14.7	18.4 ± 14.0	0.108
course of disease (year)	7.9 ± 8.4	7.0 ± 7.2	0.729
**Seizure type**			
Focal onset	19 (90.5%)	16 (80.0%)	0.410
Generalized onset	2 (9.5%)	4 (20.0%)	
**Pathogeny**			
Structure	6 (28.6%)	6 (30.0%)	0.658
Infectivity	3 (14.3%)	2 (10.0%)	
Immunity	1 (4.8%)	0 (0.0%)	
Unknown	11 (52.4%)	12 (60.0%)	
**Frequency_a_ (seizures per year)**			
0–4	13 (65.0%)	5 (26.3%)	0.044
5–12	4 (20.0%)	3 (15.8%)	
13–52	2 (10.0%)	6 (31.6%)	
>52	1 (5.0%)	5 (26.3%)	
**Frequency_b_ (seizures per year)**			
≤ 4 times	13 (65.0%)	5 (26.3%)	0.015
>4 times	7(35.0%)	14 (73.7%)	
**Number of ASMs**			
1	15 (78.9%)	6 (40.0%)	0.017
2	2 (10.5%)	8 (53.3%)	
3	2 (10.5%)	1 (6.7%)	
**Compliance**			
Good	10 (52.6%)	7 (43.8%)	0.738
Poor	9 (47.4%)	9 (56.3%)	

*Continuous variables were expressed as mean ± standard deviation, and categorical variables were expressed as percentages.*

### Characteristics of Intestinal Microbes of Epilepsy Patients in the Urban Group and Rural Group

**FIGURE 5:**
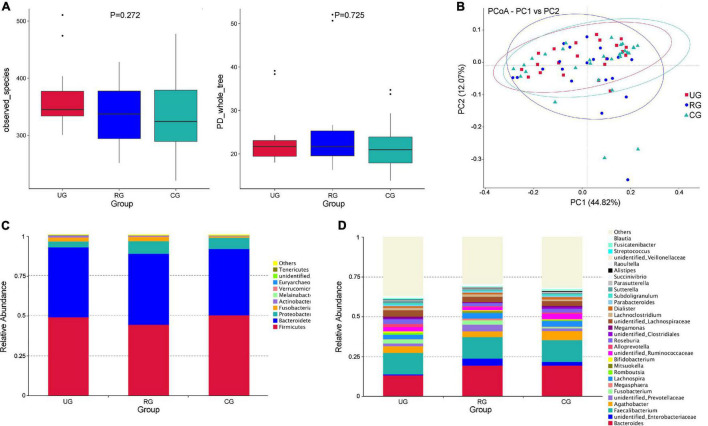
Differences of intestinal microbes among the urban group (UG), rural group (RG), and control group (CG). **(A)** The α diversity (observed_species and PD_whole_tree index) showed that there was no significant difference of the diversity among the three groups (*P* > 0.05, calculated by the Kruskal-Wallis test). **(B)** The PCoA analysis of β diversity based on the weighted UniFrac distance showed that the composition of intestinal microbe among the UG, RG, and CG was different (UG is expressed by red squares, RG is expressed by blue dots, and CG is expressed by green triangles). **(C)** The relative abundance of the top 10 microbes at the phylum level in the UG, RG and CG. **(D)** The relative abundances of the top 30 microbes at the genus level in the UG, RG and CG.

The α-diversity analysis indicated that there was no statistical difference in the intra-sample diversity of the UG, RG, and CG (*P* > 0.05), taking observed species index and phylogenetic diversity (PD whole tree) index as examples (calculated by the Kruskal–Wallis Test) (Figure 5A). PCoA analysis of β-diversity based on weighted UniFrac distances showed that the composition of intestinal microbes among the three groups was different (Figure 5B).

At the phylum level, *Firmicutes* was the largest phylum of the UG, RG, and CG. The abundance of *Firmicutes* in the RG was lower than that of the UG and CG (49% UG, 44% RG, and 50.3% CG). *Bacteroides* was the second largest phylum (43.7% UG, 44.0% RG, and 50.3% CG). The abundance of *Melainabacteria* (0.03% UG, 0.04% RG, and 0.1% CG) and *Euryarchaeota* (0.001% UG, 0.002% RG, and 0.05% CG) of the UG and RG were decreased, when compared with those of the CG (Figure 5C). At the genus level, the abundance of *Megamonas* (1.0% UG, 0.3% RG, and 0.8% CG) and *Subdoligranulum* (1.19% YG, 0.06% RG, and 1.12% DG) of the RG were more decreased than those of the UG and CG (Figure 5D).

## Discussion

Our results showed abundance difference in the epilepsy group and the control group at the genus level due to our analysis methods (16S ribosomal RNA sequencing). The results did not give absolute amounts of bacteria. We think species and ratios of bacteria were closely with epileptic activity. Therefore, we clarified changes of species and ratios of bacteria in EG and CG.

The abundance of *Fusobacteria* and *Verrucomicrobia* at the phylum level and *Alloprevotella* at the genus level were increased in EG when compared with CG. At the species level, *Ruminococcus gnavus*, *Fusobacterium mortiferumis*, and *Bacteroides fragilis* were closely related to epilepsy. Spearman’s correlation analysis further confirmed that *Fusobacterium* was significantly positively correlated with epilepsy. The random forest model prediction suggested that *Fusobacterium* played an important role in distinguishing epilepsy and healthy people. This was consistent with the research results of Şafak et al. (2020) who also found significantly increased *Fusobacteria* in 10.6% of the intestines of epilepsy patients, but not in CG. We think *Fusobacteria* might be a therapeutic target in the future.

The abundance of *Verrucomicrobia* in patients with epilepsy was increased, which was consistent with a previous study (Gong et al., 2020). *Verrucobacterium* phylum was positively correlated with serum pro-inflammatory cytokine IFNγ (Lin et al., 2019). It increased the permeability of the blood-brain barrier (BBB) and the small intestinal epithelial barrier by modifying the localization of ZO-1 and claudin-5 (Rahman et al., 2018). As a result, neuroinflammatory substances could easily enter the brain and affect brain function, which was thought to be related to the pathogenesis of epilepsy (Gao et al., 2017). *Verrucomicrobia* is also associated with the increase of glutamate and glutamine, and decrease of serotonin (Sun et al., 2016), indicating that *Verrucomicrobia* may be involved in the pathogenesis of epilepsy by affecting neurotransmitter levels.

The level of *Alloprevotella* in patients with epilepsy was increased, which was positively correlated with the occurrence of epilepsy. *Alloprevotella* can produce SCFAs and have anti-inflammatory effects (Liu et al., 2018; Wei et al., 2018), which has been negatively correlated with the level of inflammatory factors such as IL-4 and IL-10 (Li et al., 2019). IL-10 was an anti-inflammatory factor (Sanjabi et al., 2009). Recent studies showed that neuroinflammation can cause high excitability of neurons, leading to seizures (Sanz and Garcia-Gimeno, 2020). *Prevotella* may be involved in the onset of epilepsy by affecting the levels of cytokines. However, some studies showed that *Prevotella* produced short-chain fatty acids (SCFAs), which have anti-inflammatory effects (Liu et al., 2018; Wei et al., 2018), although the mechanism of action needs to be further clarified.

In addition, we found the abundance of *Ruminococcus gnavus* was significantly increased at the species level, which positively correlated with epilepsy. It *Ruminococcus gnavus* has been confirmed to be closely related to irritable bowel syndrome (Hall et al., 2017), Crohn’s disease (Nagayama et al., 2020), type 2 diabetes (Wang et al., 2020), attention deficit hyperactivity disorder (Wan et al., 2020), and generalized anxiety (Jiang et al., 2018). A previous study showed that *Ruminococcus gnavus* promoted disease progression by aggravating inflammation and inducing degradation of the intestinal mucosa (Clark and Mach, 2016), which may be one of the mechanisms of epilepsy progression.

*Fusobacterium mortiferum* was also closely related to epilepsy in our study. A study showed *Fusobacterium mortiferum* was positively correlated with serum levels of 2-deoxy-D-ribose (Liang et al., 2020). The 2-deoxy-D-ribose cooperates with thymidine phosphorylase and vascular endothelial growth factor to inhibit tight junction proteins, leading to the destruction of BBB, which results in inflammatory diseases of central nervous system (Chapouly et al., 2015). In addition, 2-deoxy-D-ribose could also induce neuronal apoptosis through oxidative stress (Formichi et al., 2009), which may be involved in the onset of epilepsy. These studies indicated that *Fusobacterium mortiferum* and its metabolites may be involved in epilepsy, but no direct evidence has yet been reported.

Correlation analysis showed that *Bacteroides fragilis* was also closely related to epilepsy, which has not been previously reported in epilepsy. *Bacteroides fragilis* was an anaerobic rod-shaped Gram-negative bacteria that could secrete pro-inflammatory substances, such as LPS and *bacteriodes fragilis* toxin, which can cause increased permeability of the BBB through E-cadherin of epithelial cells. This made it easier for microbial-derived neurotoxic substances to enter the brain and thus promoted neuronal hyper-excitability and epilepsy (Vezzani et al., 2016; Lukiw, 2020). We therefore speculate that *Bacteroides fragilis* and its metabolites related to epilepsy may induce a neuroinflammatory response by destroying BBB.

Our study also showed that the abundance of *Bacteroides dorei* in patients with epilepsy was increased at the species level. A previous study showed that the colonization of *Bacteroides dorei* in germ-free mice enhanced the accumulation of TH17 cells (Nagayama et al., 2020), which has been thought to play a central role in the pathogenesis of autoimmune epilepsy (Han et al., 2020).

PICRUSt analysis indicated that the upregulation of starch, and sucrose metabolism, and PPAR signaling pathway may be closely related to the onset of epilepsy (Eor et al., 2021). A ketogenic diet can up-regulate the abundance of PPAR in the brains of mice, and then reduce epileptic seizures (Simeone et al., 2017; Eor et al., 2021). These results indicated that PPAR pathway could be a target for the treatment of epilepsy. Studies have shown significantly increased abundance of ABC transporter in patients with drug-resistant epilepsy, when compared with patients with drug-sensitive epilepsy (Peng et al., 2018). However, in our study, the abundance of ABC transporter, as well of those of starch and sucrose metabolism, were relatively decreased in patients with epilepsy, and PPAR signaling pathway was significantly increased, which is inconsistent with previous research results. Enriched ABC transporters in the disease group is indeed more meaningful. Contrary results obtained in our study may be related to the insufficient sample size and different populations. We will enlarge the sample size and hope to further determine gut microbiome characteristics of drug-sensitive and drug-resistant epilepsy.

Based on the above results, we think the gut microbes may affect the occurrence and progress of epilepsy. There were three possible mechanisms. First, neuroinflammation played an important role in epilepsy (Yamanaka et al., 2021). Proinflammatory cytokines cause morphological changes in human-derived pericyte. Cerebrovascular pericytes undergo redistribution and remodeling contributing to blood-brain barrier (BBB) permeability. Increased BBB permeability was involved in the pathogenesis of epilepsy. Our results showed *Fusobacterium, Verrucomicrobia*, *Alloprevotella*, *Ruminococcus gnavus*, *Fusobacterium mortiferum*, *Bacteroides fragilis*, and *Bacteroides dorei* increased in patients with epilepsy. They may induce proinflammatory cytokines. Second, the increases of glutamate and glutamin were also involved in the process of epilepsy. IL-1 beta influence on glutamate levels was involved in the etiology of both epilepsy and inflammatory bowel disease (Rindflesch et al., 2018). In our study, we also found increased *Verrucomicrobia* which may be involved in the pathogenesis of epilepsy by affecting neurotransmitter levels. *Verrucomicrobia* is also associated with increases of glutamate and glutamine, and decrease of serotonin (Sun et al., 2016). Third, hippocampal neuronal apoptosis was closely with progress of epilepsy (Liu et al., 2019). Our results showed *Fusobacterium mortiferum* increased. It can promote serum levels of 2-deoxy-D-ribose which induced neuronal apoptosis through oxidative stress (Formichi et al., 2009).

Because the environment of residence can affect the composition of intestinal flora, with a higher prevalence of epilepsy in rural areas, we determined the differences of intestinal flora between urban vs. rural groups. Our results showed that the abundance of *Escherichia coli*, *Fusobacterium varium*, *Prevotella stercorea*, and *Prevotellaceae bacterium DJF VR15* were significantly increased in the RG, and the abundance of *Roseburia and Romboutsia* were increased in the UG.

Certainly, levels of gut microbiota are susceptible to many factors, such as age, gender, diet, geographical location, exercise, drug usage, metabolism, and surgery (Yang et al., 2021). Especially, ketogenic diet can control seizure attack. *Akkermansia muciniphila* and *Parabacteroides* played an essential role in the anti-seizure effect of ketogenic diet (Gómez-Eguílaz et al., 2018; Olson et al., 2018). Therefore, we also try to apply ketogenic diet to treat patients and explore the relationship of ketogenic diet with gut microbes which we have found in EG in the next experiment.

Previous studies showed that 16 representative drugs in the subgroup of the Anatomical Therapeutic Chemical classification system, including anti-seizures medication (ASMs) had no obvious antibacterial effect (Dahlin and Prast-Nielsen, 2019). Consistent with other studies, we found that ASM had no obvious effect on the patient gut microbiome; however, we realize that our sample size was such that this question was underpowered.

### Limitations

Our study had some limitations. First, although we used the 16S rRNA PICRUSt function prediction to analyze the possible mechanism of intestinal flora effects on epilepsy, this was a correlative study, and it did not present direct evidence that increased levels of certain intestinal flora resulted in epilepsy. Studies with larger sample sizes based on metagenomics, proteomics, and metabonomics should therefore be used to further characterize the mechanism of gut microbiota involvement in the pathogenesis of epilepsy. Second, we have not made a subgroup analysis of epileptic patients due to small sample size in EG, such as different etiologies, different types of epilepsies, seriousness of epileptic seizures, effect of ASM, etc. However, we can make further analysis if the sample size is sufficient.

## Conclusion

In summary, there were significant differences in the compositions of intestinal microbes between epilepsy patients and healthy controls. *Fusobacterium mortiferum*, *Bacteroides fragilis*, *Ruminococcus gnavus*, and *Fusobacterium* sp. may be potential risk factors for epilepsy, and increased levels of *Fusobacterium* may be a biomarker for the diagnosis of epilepsy. Overall, the characteristic changes of intestinal flora and metabolic pathways in patients with epilepsy suggest that it may be a new target for the treatment of epilepsy.

## Data Availability Statement

The original contributions presented in the study are included in the article/supplementary material, further inquiries can be directed to the corresponding author/s.

## Ethics Statement

The studies involving human participants were reviewed and approved by the Ethics Committee of the Affiliated Hospital of Guizhou Medical University. The approval number was 146. The patients/participants provided their written informed consent to participate in this study.

## Author Contributions

ZF and LY contributed to the drafting of the manuscript. YC, JX, and MZ contributed to analyses and interpretations of the data. LD, QZ, MW, and YY contributed to the collection of data. ZX and GW contributed to the conception and critical revision of the manuscript. All authors contributed to the article and approved the submitted version.

## Conflict of Interest

The authors declare that the research was conducted in the absence of any commercial or financial relationships that could be construed as a potential conflict of interest.

## Publisher’s Note

All claims expressed in this article are solely those of the authors and do not necessarily represent those of their affiliated organizations, or those of the publisher, the editors and the reviewers. Any product that may be evaluated in this article, or claim that may be made by its manufacturer, is not guaranteed or endorsed by the publisher.

## Footnotes

1
http://qiime.org/

2
https://drive5.com/uparse/
